# Functional Properties and Sustainability Improvement of Sourdough Bread by Lactic Acid Bacteria

**DOI:** 10.3390/microorganisms8121895

**Published:** 2020-11-30

**Authors:** Vera Fraberger, Claudia Ammer, Konrad J. Domig

**Affiliations:** Department of Food Science and Technology, BOKU–University of Natural Resources and Life Sciences Vienna, Muthgasse 18, 1190 Vienna, Austria; claudia.ammer@gmx.at (C.A.); konrad.domig@boku.ac.at (K.J.D.)

**Keywords:** sourdough, lactic acid bacteria, spoilage, molds, bacilli, γ-aminobutyric acid

## Abstract

Preventing food spoilage without the addition of chemical food additives, while increasing functional properties of wheat-based bakery products, is an increasing demand by the consumers and a challenge for the food industry. Within this study, lactic acid bacteria (LAB) isolated from sourdough were screened in vitro for the ability to utilize the typical wheat carbohydrates, for their antimicrobial and functional properties. The dual culture overlay assay revealed varying levels of inhibition against the examined fungi, with *Lactiplantibacillus plantarum* S4.2 and *Lentilactobacillus* *parabuchneri* S2.9 exhibiting the highest suppression against the indicator strains *Fusarium graminearum* MUCL43764, *Aspergillus fumigatus*, *A. flavus* MUCL11945, *A. brasiliensis* DSM1988, and *Penicillium roqueforti* DSM1079. Furthermore, the antifungal activity was shown to be attributed mainly to the activity of acids produced by LAB. The antibacillus activity was evaluated by the spot-on-the-lawn method revealing a high inhibition potential of the majority of LAB isolated from sourdough against *Bacillus cereus* DSM31, *B. licheniformis* DSM13, *B. subtilis* LMG7135, and *B. subtilis* S15.20. Furthermore, evaluating the presence of the glutamate decarboxylase gen in LAB isolates by means of PCR showed a strain dependency of a potential GABA production. Finally, due to improved functional activities, LAB isolated from sourdoughs exhibit promising characteristics for the application as natural preservatives in wheat-based bakery products.

## 1. Introduction

Within the last years, the importance of the new term “clean label” has increased due to a rising awareness of the consumer regarding healthiness and sustainability of food products [1]. Hence, the industry is investigating the potential application of natural alternatives to chemical food additives. Fermentation processing is one of the oldest technologies applied and is an effective way for bio-preservation, leading to improved sensory and nutritional qualities, and an increased microbiological safety and shelf life [2]. These reasons led to a regained momentum of sourdough products within the last years [3]. Lactic acid bacteria (LAB) dominate the sourdough microbiota and co-exist with yeasts, being present generally at a ratio of LAB to yeasts of 100:1 [4], in which the metabolism of LAB is especially considered to primarily determine the numerous functional advantages of sourdough [5]. 

The preservative effect obtained by the application of sourdough is mainly due to the production of lactic and acetic acids, which leads to a drop of the pH, with a stable sourdough exhibiting pH values of approximately 3.5 to 4.0 [6]. Furthermore, antimicrobial compounds, such as bacteriocins, ethanol, hydrogen peroxide, and fatty acids are secreted by several lactobacilli to inhibit the growth of undesired microorganisms [7]. Mold [8] and rope spoilage [9] are key limiting factors for the shelf life of bread resulting in pecuniary losses for the baking industry [10,11]. Therefore, several studies have focused on the biological control of these foodborne pathogens, showing proper antifungal and even anti-aflatoxigenic [12,13,14], as well as antibacterial potential of sourdough LAB [15]. The contaminants mainly derive from the baking environment, flour, yeast, or further ingredients [9,16] and exhibit suitable growth conditions within the bread regarding the water activity (a_w_) and pH [17]. 

In the case of rope spoilage, *Bacillus* species, such as *B. subtilis*, *B. cereus*, *B. licheniformis*, and *B. pseudomesenteroides* are assumed to be the main triggers [9,11,17,18]. *Bacillus* spores are heat resistant and survive the baking temperatures within the crumb ranging from 97–101 °C for a few minutes. The endospores germinate under the favorable conditions within the bread (a_w_ > 0.95, pH > 5) and lead to its deterioration [18]. This process starts with an unpleasant odor similar to cantaloupe [10]. Further, microbial amylases and proteases lead to the breakdown of starch and proteins, resulting in a discolored, sticky crumb, and slimy extracellular polysaccharides, in which these visible ropes appear within 12–24 h after baking [17]. 

The most common molds associated with bread spoilage belong to the genera *Aspergillus*, *Penicillium* [19], *Fusarium*, *Rhizopus*, and *Mucor* [20]. Due to the ubiquitous occurrence of the conidiospores in the biosphere and the favorable growth conditions within the bread, fungal growth is supported [8]. Several fungi are known for their potential to produce mycotoxins, which are toxic secondary metabolites, exhibiting acute or chronic toxicity, for example, cancerogenic or nephrotoxic activity [21]. 

In addition to the antimicrobial effect, LAB have been associated with the production of bioactive compounds, which exhibit an advantageous impact on the nutritional quality and sensory aspect of bakery products. In this context, GABA (γ-aminobutyric acid) is of interest. This non-protein amino acid is synthesized by the enzyme glutamate decarboxylase (GAD), which catalyzes the decarboxylation of L-glutamate to GABA [22]. The GAD system contributes to an increased bacterial acid resistance [23]. Furthermore, the presence of this amino acid is related to beneficial health effects, such as a hypotensive effect, antidiabetic, and neurotransmission, as well as depression and anxiety reduction properties due to tranquilizer effects [24,25,26]. Fermented foods have been proven to be an excellent source of GABA, in which LAB exerting the GAD system are contributing to the high concentrations in sourdough of up to 39 mg/kg [27]. 

The advantageous effects exerted by LAB on human wellbeing are known for a long time and are industrially used due to their potential to increase the safety, sensory, and stability of foods [28,29]. Furthermore, LAB have been applied for decades in food preservation, leading to their classification as generally recognized as safe (GRAS) and some of the species are harboring the qualified presumption of safety (QPS) status [30]. 

Therefore, this study aimed to screen LAB isolated from traditional Austrian type I wheat and rye and type III sourdoughs concerning their antimicrobial activities and the presence/absence of the GAD gene for further industrial applications on wheat-based bakery products. 

## 2. Materials and Methods 

### 2.1. Matrices, Producer, and Indicator Strains

LAB isolates used within this study were previously obtained from traditional Austrian wheat and rye sourdoughs as previously published by Fraberger, et al. [31] or reactivated dried wheat sourdoughs. Prior to the measurements, cryoprotected isolates (−80 °C) were revitalized in MRS broth (Merck, Darmstadt, Germany) overnight at 30 °C under anaerobic conditions. 

In total, 184 LAB isolates (producers) were screened for their ability to utilize the typical wheat carbohydrates (fructose, maltose, glucose, raffinose, sucrose, and maltodextrin) and the presence/absence of the GAD gene. Furthermore, 77 isolates were selected to analyze the potential to inhibit the growth of five fungal species. Further, 65 isolates were chosen for the evaluation of their antibacillus activity against four indicators, especially against those responsible for the spoilage of bakery products. 

Indicator strains were either purchased from DSMZ (Leibniz-Institute DSMZ-German Collection of Microorganisms and Cell Cultures; Braunschweig, Germany) or BCCM/MUCL (Belgian Coordinated Collection of Microorganisms/Agro-food and Environmental Fungal Collection; Bruxelles, Belgium) databases. The indicator strain *Bacillus subtilis* S15.20 was isolated from sourdough (Table 1). 

### 2.2. Carbohydrate Metabolism

LAB isolates, listed in Table S1, were characterized for their carbohydrate metabolism profile typically present in Austrian wheat [32]. The growth of isolates was tested on D-(-)-fructose (Merck, Darmstadt, Germany), D-(+)-raffinose (Diagonal GmbH & Co. KG, Münster, Germany), D-(+)-maltose (Sigma-Aldrich, St. Louis, MO, USA), sucrose (Merck, Darmstadt, Germany), D-(+)-glucose (Carl Roth, Karlsruhe, Germany), and maltodextrin (Sigma-Aldrich, St. Louis, MO, USA) as sole carbohydrate sources. Reactivated isolates were transferred into a 5 mL MRS broth and incubated for 24 h at 30 °C under anaerobic conditions (N_2_ 85%, H_2_ 5%, CO_2_ 10%). Further, the MRS broth lacking carbohydrates (condalab, Madrid, Spain) was inoculated with 2% (*v*/*v*) of the corresponding culture and incubated for 48 h at 30 °C under anaerobic conditions. One hundred sixty microliters of the carbohydrate media (MRS broth with 2% (*v*/*v*) of the corresponding carbohydrate) were transferred into 96-well plates and inoculated with 3.2 µL of bacterial suspension. Wells were overlaid with paraffin oil (biomerieux, Marcy l’Etoile, France) to generate anaerobic conditions and incubation was carried out at 30 °C. 

Prior to the measurements, plates were shaken for 10 min at 30 °C for 1450 rpm using a bioShake iQ (Q instruments, Jena, Germany). Afterwards, the optical density at 620 nm (OD_620_) was examined in a plate photometer (Tecan Sunrise, Männedorf, Switzerland) for up to 7 days in duplicates. 

### 2.3. Determination of Antifungal Activity by the Dual Culture Overlay Assay

The dual culture overlay assay (illustrated in Figure S1a) was performed to evaluate the antifungal activity of 77 LAB isolates (producers) for their potential to inhibit the growth of five fungal species (indicators). LAB isolates belonged to species *Levilactobacillus* (*Lev.*) *brevis*, *Lev. hammesii*, *Lev. senmaizukei*, *Lev. spicheri*, *Loigolactobacillus* (*Lo.*) *coryniformis*, *Latilactobacillus* (*La*.) *curvatus*, *Lentilactobacillus* (*Len.*) *diolivorans*, *Len. kisonensis*, *Len. otakiensis*, *Len. parabuchneri*, *Companilactobacillus* (*Co.*) *kimchii*, *Co. paralimentarius*, *Lactiplantibacillus* (*Lp.*) *plantarum*, *Lp. xiangfangensis*, *Limosilactobacillus* (*Li.*) *pontis*, *Furfurilactobacillus* (*Fu.*) *rossiae*, *Latilactobacillus* (*Lat.*) *sakei*, *Fructilactobacillus* (*Fr.*) *sanfranciscensis*, *Lacticaseibacillus* (*L.*) *paracasei*, *Paucilactobacillus* (*Pa.*) *vaccinostercus*, *Pediococcus parvulus*, *P. pentosaceus*, *Weissella cibaria,* and *W. viridescens*. 

**Figure 1:**
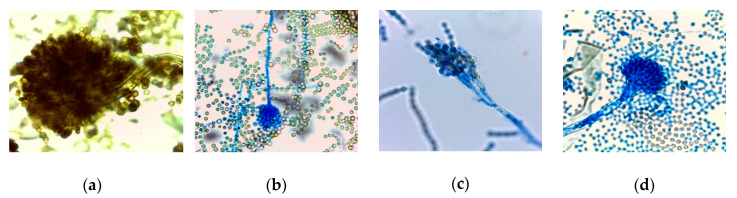
Asexual reproduction structures of lactophenol cotton blue stained fungal species examined by a light microscope with a total magnification of 1000x; (**a**) *Aspergillus brasiliensis* DSM1988, (**b**) *A. flavus* MUCL11945, (**c**) *Penicillium roqueforti* DSM1079, (**d**) *Aspergillus fumigatus*.

Fungal species *Aspergillus fumigatus*, *A. brasiliensis* DSM1988, *A. flavus* MUCL11945, *Penicillium roqueforti* DSM1079, and *Gibberella zeae* (anamorph *Fusarium graminearum*) MUCL43764 (Figure 1) were selected as they are either known to produce mycotoxins or are commonly isolated from contaminated baked goods. The method was performed according to Magnusson and Schnurer [33] and Manini, et al. [34] with some modifications. Briefly, the fungal spore suspensions were prepared by growing the molds on a Sabouraud-1% glucose agar (Dinkelberg Analytics, Gablingen, Germany) for 7 days at 25 °C. Afterwards, conidia were collected by adding 25 mL of sterile buffered peptone water (Merck, Darmstadt, Germany) to the inoculated agar, horizontally shaking for 10 min at 50 rpm, and inoculating 10 mL of malt extract soft agar with 1 mL of a fungal spore-suspension. 

Five milliliters of MRS broth were inoculated with the corresponding LAB isolate and incubated for 24 h at 30 °C under anaerobic conditions. Further, bacteria were transferred onto the corresponding agar in 2 cm single lines. After incubation for 48 h, the agar was overlaid with 10 mL of malt extract soft agar inoculated with fungal spores (4 to 5 log_10_ CFU mL^−1^). After incubation at 30 °C for 48 h, the plates were examined for clear zones around the bacterial lines. The experiment was performed in triplicates. The degree of inhibition was defined as very strong (+++; Ø > 8 mm), intermediate (++; 8 ≤ Ø < 2 mm), weak (+; Ø ≤ 2 mm), and no inhibition (-). 

To evaluate the synergistic effect of the sodium acetate and the present LAB, isolates exhibiting a very strong inhibition effect, were further tested on an APT agar (Oxoid Ltd., Basingstoke, UK). This medium is especially used for the cultivation of heterofermentative LAB lacking, compared to the MRS agar, sodium acetate, which was determined to have a strong antifungal property itself [35]. 

### 2.4. Determination of Antibacillus Activity by Spot-on-the-Lawn Technique

The antibacillus activity of 65 LAB isolates was tested against *B. cereus* DSM31, *B. licheniformis* DSM13, *B. subtilis* LMG7135, and the sourdough isolate *B. subtilis* S15.20 in triplicates according to Tremonte, et al. [36] with some modifications. Briefly, the spot-on-the-lawn technique was applied by inoculating Plate Count agar (PCA; Merck, Darmstadt, Germany) plates with spots of 15 µL bacterial overnight cultures followed by an incubation for 24 h at 30 °C under anaerobic conditions. Each indicator strain was transferred into 10 mL soft agar (2% *v*/*v*), before pouring onto PCA plates, on which LAB isolates were grown. After 24 h at 30 °C, the plates were examined for inhibition zones around the spots. The degree of inhibition was defined as low (4 ≤ Ø < 12 mm), moderate (12 ≤ Ø < 20 mm), strong (20 ≤ Ø < 28 mm), and very strong (Ø ≥ 28 mm). 

### 2.5. Screening of LAB Strains for the Presence of Glutamic Acid Decarboxylase Gene 

The presence/absence of the GAD gene within 179 LAB isolates was evaluated according to Demirbaş, et al. [37]. The bacterial genomic DNA of isolates was isolated as described by Fraberger, Unger, Kummer and Domig [31]. Each PCR mixture (25 µL) contained 12.5 µL of AccuStart II PCR ToughMix (QuantaBio, Beverly, MA, USA) and 20 mM of each primer CoreF (5′-CCTCGAGAAGC CGATCGCTTAGTTCG-3′) and CoreR (5′-TCATATTGACCGGTATAAGTGATGCCC-3′) [22]. The PCR program was as follows: 95 °C for 2 min, 20 cycles of 95 °C for 30 s, 60 °C for 20 s and 72 °C for 30 s, followed by a final extension step of 72 °C for 5 min. Afterwards, PCR products were separated with electrophoresis on 2% (*w*/*v*) agarose-gels at 90 V for 1.5 h before staining with GelRed (Biotium, Fremont, CA, USA) and visualized under UV-light. The presence of the GAD gene was indicated by an amplicon size of approximately 540 bp.

### 2.6. Lactic Acid Bacteria Strain Differentiation 

The repetitive element PCR (rep-PCR) was applied for the genomic characterization of LAB (Table S2) using the single nucleotide primer (GTG)_5_ (5′-GTGGTGGTGGTGGTG-3′) [38,39]. Amplification was carried out on a Mastercycler nexus SX1 (Eppendorf, Germany) with reaction mixtures (25 µL) containing 12.5 µL AccuStart II PCR ToughMix (QuantaBio, USA), 11 µL ultra-high-quality water, 1 µL of primer (50 pmol/µL), and 0.5 µL of the DNA template. The following PCR conditions were used: Initial denaturation for 7 min at 94 °C, followed by 30 cycles of 90 °C for 30 s, 40 °C for 1 min and 65 °C for 8 min, and a final extension of 65 °C for 16 min. PCR products were electrophoresed in a 2% agarose-gel for 1 h and 50 min before staining with GelRed (Biotium, USA) and visualization under UV light. Resulting fingerprint files were analyzed by the BioNumerics V.8.0 software package and the average linkage (UPGMA) dendogram for LAB was constructed as described by Gevers, Huys and Swings [39].

### 2.7. Statistical Analysis

The statistical analysis was performed using the IBM SPSS Statistics 24.0. The results for the anti-bacillus activity were expressed as the mean (*n* = 3) ± standard deviation (SD). Data were examined using the one-way analysis of variance (ANOVA). The correlation analysis was performed between the carbohydrate metabolism of LAB and antifungal activity. 

## 3. Results and Discussion

### 3.1. Strain Differentiation

**Figure 2:**
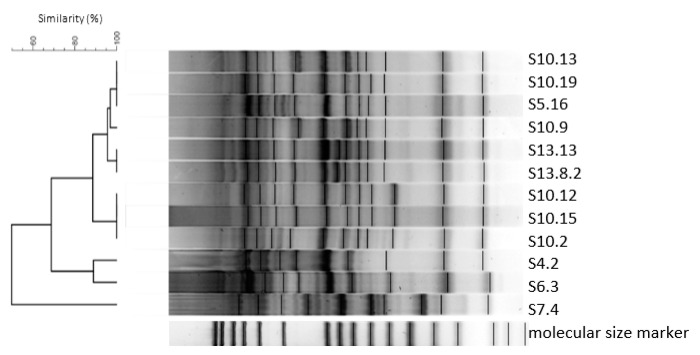
Dendogram based on the cluster analysis of (GTG)_5_-PCR fingerprints from *Lactiplantibacillus plantarum* isolated from sourdough.

In total, 171 LAB isolates of 28 different species were examined by rep-PCR using the (GTG)_5_ primer. A detailed list of the number of isolates per species and isolate code is illustrated in Table S2. Differences on the strain level were observed between isolates within the species *Enterococcus hirae*, *Levilactobacillus brevis*, *Lev. senmaizukei, Loigolactobacillus coryniformis*, *Latilactobacillus curvatus*, *Lacticaseibacillus paracasei*, *Lactiplantibacillus plantarum*, *Fructilactobacillus sanfranciscensis*, and *Weissella cibaria*. As exemplary illustrated for the species *Lp. plantarum* (Figure 2), the (GTG)_5_-PCR fingerprinting and cluster analysis separated the analyzed strains into seven groups according to their similarity. Strains S10.13, S10.19, and S5.16 showed 100% similarity. This result is reflected to those received for the antimicrobial activity and absence/presence of the GAD gene. 

### 3.2. Carbohydrate Metabolism

The carbohydrate fermentation profile of LAB isolates regarding wheat relevant carbohydrates [32] was evaluated as LAB cell counts and, therefore, sensory and functional attributes are depending on the present strains and available carbohydrates. The metabolism profiles are illustrated in Table S1. According to our results, the tested microorganisms favored glucose, maltose, and fructose as these sole carbon sources were utilized by 99%, 94%, and 89% of the tested isolates, respectively. These data were in accordance with the information received by Manini, Casiraghi, Poutanen, Brasca, Erba and Plumed-Ferrer [34] as seven typical sourdough species analyzed regarding their carbohydrate fermentation profile showed the fermentation of D-glucose, D-fructose, and D-maltose. Regarding *Enterococcus* strains, the majority exhibited growth on each of the tested carbohydrates, except for TS4.8, which did not grow on sucrose, and TS4.1, which exhibited no growth on raffinose. Concerning *Leuconostoc* spp., all tested strains metabolized the applied sugars, except for TS2.8, which did not utilize raffinose as a sole carbohydrate. *P. pentosaceus* strains fermented fructose, glucose, and maltose, except for isolate S10.10, which could not utilize fructose. Variable fermentation profiles were obtained for 38% of *P. pentosaceus* isolates due to the inability to grow on maltodextrin, raffinose, or sucrose.

All examined lactobacilli except for *Fr. sanfranciscensis* TS7.3 and *Len. otakiensis* S3.11 exhibited a very strong or strong growth on D-(+)-glucose as a sole carbohydrate source. The species *Pa. vaccinostercus*, *Co. paralimentarius,* and *Lat. sakei*, as well as the isolate *Len. otakiensis* S3.1 showed no growth on D-(+)-maltose. However, other tested *Len. otakiensis* isolates (S3.11, S3.15) were able to grow. D-(-)-fructose as a sole carbohydrate source was not metabolized by any isolates belonging to the species *Co. kimchii*, *Co. paralimentarius*, *Fr. sanfranciscensis*, and *Pa. vaccinostercus*. Further, *Lp. plantarum* S7.4 was not able to ferment fructose. The growth of lactobacilli on maltodextrin as a sole carbohydrate resulted in either poor or no growth of 68% of the tested isolates, which was even evaluated by Watson, et al. [40]. The tested *Lev. brevis* isolates and the majority of *Lp. plantarum* strains (78%) metabolized maltodextrin. Only one strain of *La. curvatus* and *Lo. coryniformis* exhibited strong growth. Within the species *La. curvatus*, isolates TS4.11 and S5.7.1 were the only tested ones exhibiting the potential to strongly grow on maltodextrin. Raffinose was metabolized by 46%, and sucrose by 84% of the tested LAB isolates. These results showed a high variability within the tested lactobacilli, which derived from its high phylogenetic diversity [41]. 

Due to the examination of preferred carbon sources, even differences on the strain level were observed. Fructose belongs to the group of FODMAPs (fermentable oligo-, di-, monosaccharides, and polyols), being an important player in the clinical picture of NCWS (non-celiac-wheat-sensitivity). Furthermore, it is a metabolic product of fructan, which is assumed to be the main trigger of NCWS. This study showed the potential of the majority of sourdough LAB to degrade fructose. Therefore, a synergistic effect with specific fructan degrading yeasts [42] could lead to well-tolerated bakery products.

### 3.3. Antifungal Activity

The molds evaluated in this study, *Aspergillus brasiliensis*, *A. fumigatus*, *A. flavus*, *Penicillium roqueforti*, and *Fusarium graminearum*, can contaminate several agricultural products and are capable of producing mycotoxins and allergenic spores, decreasing the nutritional value, and leading to economic losses [43,44,45]. Therefore, technological, economic, and health reasons lead to the necessity of bakery products exhibiting a high antimicrobial activity, in which consumers’ demand is going towards less application of chemical preservatives. To compensate the use of, for example, propionic, benzoic, and sorbic acids and some of their salts, research on the applicability of sourdough as a natural alternative was addressed by several studies [8,45,46,47]. Hence, the antifungal activity of LAB strains typically present in sourdough was evaluated against *Aspergillus fumigatus*, *A. flavus*, *A. brasiliensis*, *Penicillium roqueforti*, and *Fusarium graminearum*. LAB exhibited varying levels of inhibition against the five fungal indicator strains. In turn, the fungi displayed a different degree of sensitivity against the producer strains. Table 2 illustrates the results obtained by determining the antifungal activity using MRS and APT agar. An examination of MRS agar plates showed that *F. graminearum* MUCL43764 exhibited the highest sensitivity, being very strongly inhibited (+++; Ø > 8 mm) by 96% of the tested LAB isolates, followed by *A. fumigatus* with 55% of LAB showing a very strong inhibition potential. *Pe. roqueforti* DSM1079 manifested the highest resistance, as 65% of the LAB isolates did not alter the growth capability of this fungus, followed by *A. flavus* MUCL11945 (28%). 

Results showed the potential to inhibit mold growth for the majority of the LAB isolates tested. However, the intensity was highly dependent on the LAB strain and the fungal species. Already, Gerez, Torino, Rollán and Font de Valdez [45] revealed the strain dependency on the inhibition of fungal growth. Schwenninger, et al. [48] examined the inhibitory potential of lactobacilli against *Penicillium* sp., showing strain differences regarding the acid production of species belonging to the former *L. casei* group. In the present study, isolates of the species *Lev. brevis*, *Lo. coryniformis*, *Len. parabuchneri*, *Lp. plantarum*, *Fu. rossiae*, *Fr. sanfranciscensis*, *L. paracasei*, *Lev. senmaizukei*, *Lev. spicheri,* and *P. pentosaceus* revealed a different inhibition potential against *Pe. roqueforti* DSM1079. A very strong inhibition was observed for *Lev. brevis* S4.5, *Lo. coryniformis* S4.4.2, *Lp. plantarum* S4.2, *Len. parabuchneri* S2.9, and *L. paracasei* S9.11, whereas *Lev. brevis* S13.18, S14.3, and S6.13 did not inhibit the growth of *Pe. roqueforti* DSM1079. In contrast to our study, Magnusson, et al. [49] did not prove an inhibitory effect against *Pe. roqueforti*.

Within this study, *Lp. plantarum* exhibited the highest antifungal potential against the indicator strains. This species is known for the production of PLA, p-hydroxyphenyllactic acid, palmitic acid [50], and low molecular peptides [51] exhibiting an antifungal activity. Studies on the inhibition potential of *Lp. plantarum* in bread revealed a complete inhibition of *Fusarium* sp. and a reduced growth of *Pe. roqueforti* and *A. niger* on the contaminated bread [52]. Furthermore, a positive effect of *Lp. plantarum* and even *L. paracasei* strains towards the reduction of mycotoxins has already been observed by Bartkiene, et al. [53]. Within our study, each tested *L. paracasei* strain exhibited a very strong inhibition against *F. graminearum* MUCL43764, which was observed even by Hassan and Bullerman [54]. 

*Lp. plantarum* S4.2 and *Len. parabuchneri* S2.9 exhibited the highest inhibition (very strong or intermediate) against the indicator strains when using the MRS medium. As observable for the analyzed species, differences of inhibition were determined, leading to the conclusion that the antifungal activity was mainly strain dependent. For example, *Lev. brevis* S13.18 and S14.3 did not inhibit the growth of *Pe. roqueforti* DSM1079. However, *Lev. brevis* S3.5 exhibited intermediate and *Lev. brevis* S4.5 an even strong inhibition potential. 

Repeating the series of experiments on the APT medium showed a highly decreased activity by all isolates. The tested LAB isolates did not show any inhibitory effect against *A. brasiliensis* DSM1988 and *A. flavus* MUCL11945. *Fr. sanfranciscensis* S11.7 and S18.5 exhibited a weak degree of inhibition (0.5 to 1 mm) against *A. fumigatus*. *Pe. roqueforti* DSM1079 was inhibited only by *Lp. plantarum* S10.2 (intermediate), S10.12 and S10.14 (weak), *Lev. hammesii* S14.1 and *W. cibaria* S10.4 exhibited an intermediate inhibition. As already observable on MRS agar, *F. graminearum* exhibited the highest sensitivity also on the APT medium. Overall, results strongly suggest that the MRS medium exhibits superior properties to stimulate the production of antifungal compounds. This fact was observed even by Gajbhiye and Kapadnis [55], who concluded that glucose optimized the antifungal activity. Furthermore, within this study, a potential correlation between carbohydrates as sole carbon sources and the antifungal activity of the tested LAB strains was observable for glucose, fructose, sucrose, and raffinose regarding the inhibition of *A. fumigatus*, as well as the tested carbohydrates and the inhibition of *A. brasiliensis* DSM1988 and *Pe. roqueforti* DSM1079 (Table S3). Further, Gerez, Torino, Rollán and Font de Valdez [45] investigated the major antifungal compounds of *Lp. plantarum*, *Li. reuteri*, and *Lev. brevis* strains inhibiting fungal growth in bread. Results within this study revealed acetic and phenyllactic acids (PLA) as main antifungal components. The efficiency of organic acids was dependent on the pH values. Due to a lower pH value, the rate of a more hydrophobic, undissociated form of lactic acid increased. This form can easily enter the cell by crossing the cell membrane, releasing H^+^-ions and acidifying the cytoplasm. Hence, leading to the loss of viability and demolition of the cell [45,56].

### 3.4. Antibacilli Activity

**Figure 3:**
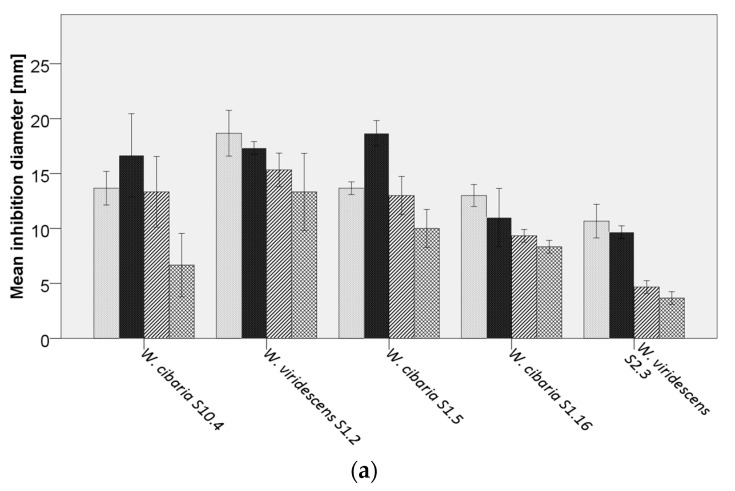

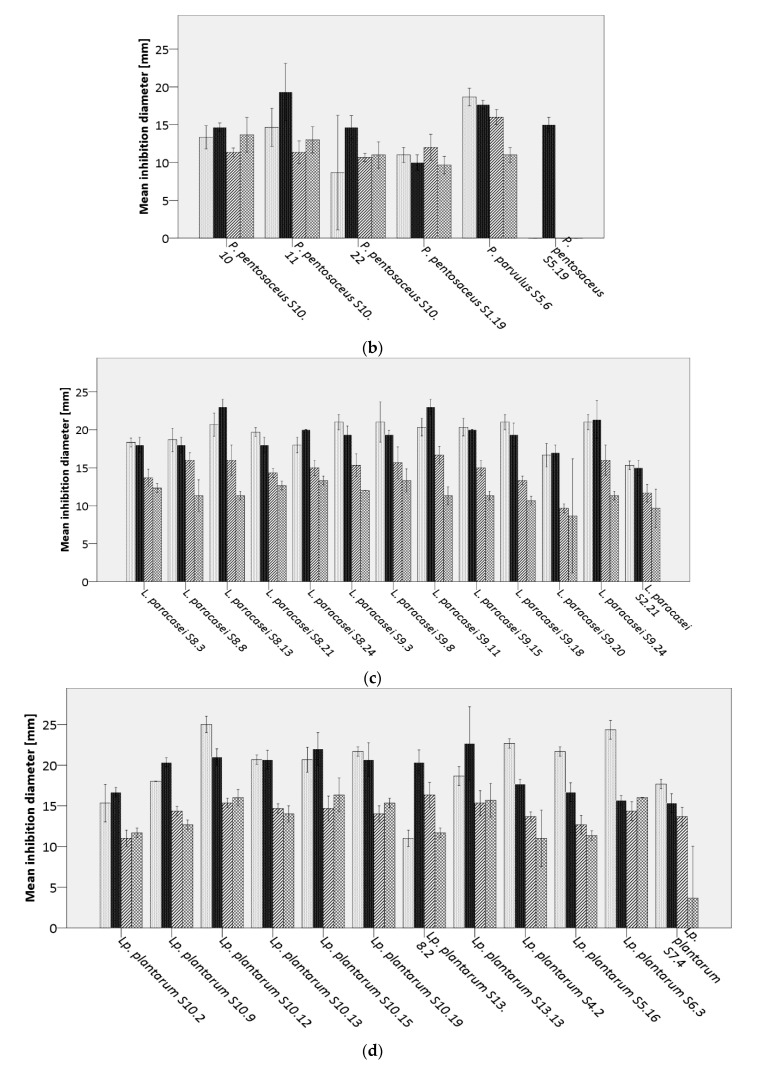

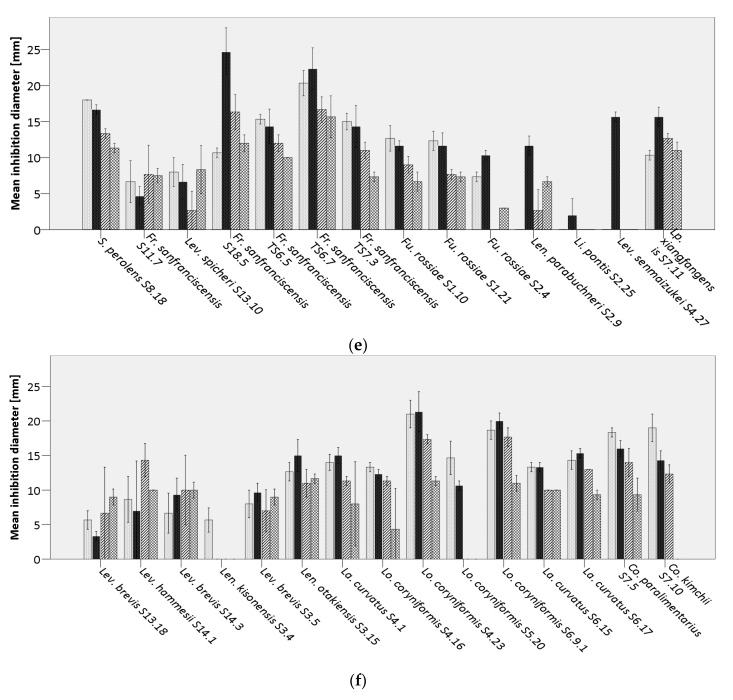
Average inhibition of analyzed lactic acid bacteria (LAB) against 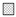 *Bacillus cereus* DSM31, 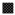 *B. licheniformis* DSM13, 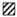 *B. subtilis* LMG7135, and 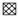 *B. subtilis* S15.20, by (**a**) *Weissella* spp., (**b**) *Pediococcus* spp., (**c**) *Lacticaseibacillus paracasei*, (**d**) *Lactiplantibacillus plantarum*, (**e**,**f**) further lactobacilli.

Bacilli are important spoilage organisms in bakery products as previous studies showed the development of rope spoilage, leading to a fruity odor after 12 h [57]. Therefore, the objective of this study was to investigate in vitro the potential of 65 LAB isolated from sourdough to inhibit spoilage bacilli and, hence, be used as future bio preservatives in wheat-based bakery products. Results showed varying levels of inhibition against the four tested indicator strains (Figure 3a–f). In turn, the bacilli displayed a different degree of sensitivity against the LAB isolates, in which the sourdough isolate *B. subtilis* S15.20 displayed the highest resistance against inhibition (11%), compared to the strain *B. subtilis* LMG7135 exhibiting resistance only against 9% of the tested isolates. Moreover, 94% of LAB inhibited the growth of *B. cereus* DSM31, and 98% showed activity against *B. licheniformis* DSM13. In addition to *L. paracasei*, *Lp. plantarum* strains showed increased inhibitory effects against bacilli. However, the ability of a species to suppress the growth of *Bacillus* spp. was shown to be strain dependent. These data are in accordance with results reported by Arena, et al. [58], in which the production of antimicrobial compounds varied between *Lp. plantarum* strains. In general, *Lp. plantarum* S6.3 and S10.12 exhibited the highest inhibitory capability (ø 23 mm) against *B. cereus* DSM31. *L. paracasei* S8.13 and S9.11, *Lp. plantarum* S13.13, and *Fr. sanfranciscensis* S18.5 showed a strong inhibition (ø 23 mm) against *B. licheniformis* DSM13. *B. subtilis* strains were especially sensitive (ø 17 mm) against *Lo. coryniformis* S6.9.1 and S4.23, as well as *Fr. sanfranciscensis* TS6.7 and S18.5. *B. subtilis* S15.20 against *Lp. plantarum* S10.15. 

Already, Mantzourani, et al. [59] tested the capacity of *L. paracasei* strains to delay the growth of *Bacillus* spp. in bread samples, showing a later appearance of rope spoilage in sourdough breads prepared with the addition of *L. paracasei*, compared to the control breads. The results derived within our study, are in accordance with the previous mentioned investigation [59], as a strong inhibitory effect of the tested *L. paracasei* strains against bacilli strains was observable. Lactic and acetic acid belong to the main inhibitory substances produced by LAB. However, further antimicrobials such as hydrogen peroxide, diacetyl, bacteriocins, and bacteriocin-like inhibitory substances (BLIS) can play important roles in inhibiting *Bacillus* spp. [60].

### 3.5. Determination of the GAD Gene

The most common way by many LAB to produce GABA is via the glutamic acid decarboxylase (GAD) system [23]. Therefore, sourdough isolates were screened for the presence of the GAD gene. Within our study, 25% of the tested isolates gave a PCR product of approximately 540 bp, including strains of *Lev. brevis*, *Lo. coryniformis*, *Lp. plantarum*, *Lev. senmaizukei*, *Lev. hammesii*, and *L. paracasei*. Further, a strain-dependency was observed for the presence of the GAD gene. For instance, only isolate *Lo. coryniformis* S4.4.2 gave a band with the primer set CoreF and CoreR. Compared to the outcomes of the study performed by Wu, Tun, Law, Khafipour and Shah [23] a strain-dependent presence of the GAD gene was even observed for *Lev. brevis*. Due to the beneficial physiological functions of GABA on human health, this bioactive component is extensively used for producing functional foods and its concentration has already been reported in several fermented foods [26]. Our results demonstrated that only part of the sourdough isolates exhibit the GAD gene, and hence a potential to produce GABA, which was even demonstrated by Villegas, et al. [61]. However, a further analysis is necessary to clearly state a possible GABA production and further quantify the yield obtained by the application of specific LAB isolates. Hence, this study is the starting point to determine isolates, which could contribute to the development of fermented foods with increased functional properties.

## 4. Conclusions

In this study, LAB strains isolated from sourdough were characterized regarding their carbohydrate metabolism, antimicrobial potential, and presence/absence of the GAD gene. Interestingly, most LAB strains exhibited an inhibitory potential against the tested molds and bacilli. However, the main antifungal components within this study still remain unknown. Therefore, further studies are necessary. The preferred carbon source within the tested lactobacilli reflected its high phylogenetic diversity. Further, a strain-dependent utilization was observed. Although there was no unique LAB strain possessing the GAD gene and the highest inhibitory potential, further studies could allow the assessment of the safe use of single or combined LAB isolates as (i) food bio-preservatives, (ii) food fermentation starters, and (iii) co-players in producing enhanced functional properties and high-quality low FODMAPs bakery products of improved quality.

## Figures and Tables

**Table 1:** Information of strains used for the determination of antifungal and antibacillus activity.

Species	Strain	Source of Isolation
*Bacillus cereus*	DSM ^1^ 31	unknown
*Bacillus licheniformis*	DSM ^1^ 13	unknown
*Bacillus subtilis*	DSM ^1^ 7135	unknown
*Bacillus subtilis*	isolate S15.20	sourdough
*Aspergillus flavus*	MUCL ^2^ 11945	wheat flour
*Aspergillus fumigatus*	n.d. ^3^	unknown
*Aspergillus brasiliensis*	DSM ^1^ 1988	*Vaccinium* sp., fruit
*Penicillium roqueforti*	DSM ^1^ 1079	gorgonzola cheese
*Gibberella zeae* [anamorph *Fusarium graminearum*]	MUCL ^2^ 43764	wheat before malting

^1^ DSMZ: Leibniz-Institute DSMZ-German Collection of Microorganisms and Cell Cultures; ^2^ BCCM/MUCL: Belgian Coordinated Collection of Microorganisms/Agro-food and Environmental Fungal Collection; ^3^ n.d.: No data available.

**Table 2:** Antifungal activity of lactic acid bacteria exerted on MRS (de MAN, ROGOSA and SHARPE; left of the vertical bar) and APT (all-purpose tween; right of the vertical bar) medium against *Aspergillus brasiliensis* DSM1988, *A. fumigatus*, *Penicillium roqueforti* DSM1079, *Fusarium graminearum* MUCL43764, and *A. flavus* MUCL11945.

Antifungal Performance on MRS|APT Medium ^1^											
Isolate	*A. brasiliensis* DSM1988	*A. fumigatus*	*Pe. roqueforti* DSM1079	*F. graminearum* MUCL43764	*A. flavus* MUCL11945	Isolate	*A. brasiliensis* DSM1988	*A. fumigatus*	*Pe. roqueforti* DSM1079	*F. graminearum* MUCL43764	*A. flavus* MUCL11945
*Co. paralimentarius* S7.5	+|-	+++|-	-|-	+++|-	-|-	*Len. parabuchneri* S2.16	++|-	+++|-	+|-	+++|+	++|-
*Fr. sanfranciscensis* S18.5	+++|-	+++|+	+|-	+++|+++	++|-	*Len. parabuchneri* S2.9	+++|-	+++|-	+++|-	+++|++	++|-
*Fr. sanfranciscensis* TS6.5	-|-	-|-	-|-	+++|-	-|-	*Lev. brevis* S13.18	++|-	+++|-	-|-	+++|-	++|-
*Fr. sanfranciscensis* TS6.7	-|-	-|-	-|-	+++|-	-|-	*Lev. brevis* S14.3	++|-	+++|-	-|-	+++|++	+|-
*Fr. sanfranciscensis* TS7.3	-|-	+|-	-|-	+++|+++	++|-	*Lev. brevis* S3.5	+++|-	+++|-	++|-	+++|+++	-|-
*Fr. sanfranciscensis* S11.7	-|-	-|+	-|-	+++|+++	++|-	*Lev. brevis* S4.5	++|-	+++|-	+++|-	+++|+++	-|-
*Fu. rossiae* S1.10	++|-	+++|-	+|-	+++|++	++|-	*Lev. brevis* S6.13	+++|-	++|-	-|-	+++|+	-|-
*Fu. rossiae* S1.21	+|-	++|-	-|-	+++|+++	+|-	*Lev. hammesii* S14.1	++|-	+++|-	+|++	+++|++	++|-
*Fu. rossiae* S2.4	++|-	+++|-	++|-	+++|+++	+|-	*Lev. kimchii* S7.10	+|-	+|-	-|-	+++|+++	+|-
*La. curvatus* S4.1	+|-	+|-	-|-	n.a.^2^	n.a.^2^	*Lev. senmaizukei* S4.24	++|-	++|-	-|-	+++|+++	++|-
*La. curvatus* S4.14	-|-	-|-	-|-	+++|-	-|-	*Lev. senmaizukei* S4.27	+++|-	+++|-	-|-	+++|+++	+|-
*La. curvatus* S5.22	-|-	-|-	-|-	+++|-	-|-	*Lev. senmaizukei* S5.18	+|-	+++|-	++|-	+++|+++	+|-
*La. curvatus* S6.15	-|-	-|-	-|-	+++|-	-|-	*Lev. senmaizukei* S6.21	++|-	++|-	-|-	+++|+++	+|-
*L. paracasei* S2.21	+|-	-|-	-|-	+++|-	-|-	*Lev. spicheri* S4.26	++|-	+++|-	-|-	+++|+++	++|-
*L. paracasei* S8.13	+|-	+++|-	+|-	+++|-	++|-	*Lev. spicheri* S6.10	+|-	++|-	-|-	+++|+	+|-
*L. paracasei* S8.18	-|-	-|-	+|-	+++|+	-|-	*Lev*. *spicheri* S13.10	++|-	+++|-	++|-	+++|+++	+|-
*L. paracasei* S8.21	++|-	+++|-	-|-	+++|++	+|-	*Li. pontis* S15.14	-|-	-|-	-|-	-|-	-|-
*L. paracasei* S8.24	++|-	++|-	-|-	+++|-	+|-	*Li. pontis* S15.3	-|-	-|-	-|-	-|-	-|-
*L. paracasei* S8.3	++|-	+++|-	-|-	+++|-	++|-	*Li. pontis* S2.25	++|-	+++|-	-|-	+++|+++	++|-
*L. paracasei* S8.8	++|-	++|-	-|-	+++|-	+|-	*Lo. coryniformis* S4.16	++|-	+++|-	-|-	+++|+	++|-
*L. paracasei* S9.11	-|-	+|-	+++|-	+++|-	+|-	*Lo. coryniformis* S4.23	+|-	++|-	-|-	+++|++	-|-
*L. paracasei* S9.15	+|-	+++|-	-|-	+++|-	+|-	*Lo. coryniformis* S4.4.2	++|-	++|-	+++|-	n.a.^2^|+++	n.a.^2^
*L. paracasei* S9.18	+|-	+++|-	-|-	+++|+	+|-	*Lo. coryniformis* S5.20	+|-	++|-	-|-	+++|++	+|-
*L. paracasei* S9.20	+|-	++|-	-|-	+++|+	++|-	*Lo. coryniformis* S6.9.1	-|-	++|-	-|-	+++|++	+|-
*L*. *paracasei* S9.24	-|-	+++|-	-|-	+++|-	+|-	*Lp. plantarum* S13.8.2	+++|-	+++|-	+|-	+++|-	-|-
*L. paracasei* S9.3	-|-	++|-	-|-	+++|-	++|-	*Lp. plantarum* S4.2	+++|-	+++|-	+++|-	+++|+++	++|-
*L. paracasei* S9.8	-|-	+++|-	-|-	+++|+	+|-	*Lp. plantarum* S5.16	+|-	+++|-	-|-	+++|++	++|-
*Lat. sakei* S4.19	-|-	-|-	-|-	+++|-	-|-	*Lp. plantarum* S6.3	++|-	+++|-	+|-	+++|+++	+|-
*Len. diolivorans* S3.2	+|-	+|-	+|-	+++|++	+|-	*Lp. plantarum* S7.4	-|-	+++|-	-|-	+++|-	-|-
*Len. kisonensis* S3.10	+|-	++|-	+|-	+++|++	+|-	*Lp. plantarum* S10.12	++|-	+++|-	+|+	+++|++	++|-
*Len. kisonensis* S3.4	+++|-	+++|-	+|-	+++|++	++|-	*Lp. plantarum* S10.13	+|-	+++|-	++|-	+++|++	++|-
*Len. otakiensis* S3.15	-|-	-|-	-|-	+++|-	-|-	*Lp. plantarum* S10.15	+|-	+++|-	+|+	+++|+++	++|-
*Lp. plantarum* S10.19	+|-	+++|-	-|-	+++|++	++|-	*P. pentosaceus* S10.10	++|-	+++|-	+|-	+++|++	-|-
*Lp. plantarum* S10.2	++|-	+++|-	-|++	+++|++	+|-	*P. pentosaceus* S10.11	++|-	+++|-	+|-	+++|+	+|-
*Lp. plantarum* S10.9	++|-	+++|-	+|-	+++|++	+|-	*P. pentosaceus* S10.22	++|-	++|-	-|-	+++|++	++|-
*Lp. plantarum* S13.13	+++|-	+++|-	+|-	+++|+++	++|-	*P. pentosaceus* S5.19	++|-	++|-	-|-	+++|+++	+|-
*Lp. xiangfangensis* S7.11	++|-	+++|-	-|-	+++|++	+|-	*W. cibaria* S10.4	++|-	+++|-	-|++	+++|++	+|-
*Pa. vaccinostercus* S6.20	-|-	-|-	-|-	-|-	-|-	*W. viridescens* S2.3	++|-	+++|-	-|-	+++|+++	++|-

^1^ Inhibition was scored as follows: +++ Very strong inhibition with large clear halos (Ø > 8 mm) around the colonies; ++ intermediate inhibition with small clear halos (8 ≤ Ø < 2 mm) around the spots; + weak inhibition around spots (Ø ≤ 2 mm); - no inhibition; ^2^ n.a.: Not analyzed.

## Supplementary Materials

The following are available online at https://www.mdpi.com/2076-2607/8/12/1895/s1. Figure S1: Evaluation of antimicrobial potential of *Loigolactobacillus coryniformis* S4.23 against (a) *Aspergillus fumigatus* using the cultural overlay assay, and (b) *Bacillus cereus* DSM31 using the spot-on-the-lawn technique; Table S1: Carbohydrate metabolism of lactic acid bacteria isolated from sourdough evaluated by OD_620_ measurements; Table S2. LAB isolates used for genomic strain differentiation by the repetitive element PCR; Table S3: Correlation coefficients of growth applying different carbohydrate sources on the inhibition of fungal activity.
