# Supplementary material for: Functional Properties and Sustainability Improvement of Sourdough Bread by Lactic Acid Bacteria
Source: Microorganisms. 2020 Nov 30;8(12):1895. doi: 10.3390/microorganisms8121895 (PMC7760938; doi:10.3390/microorganisms8121895)
Supplement: Supplementary file 1 [file microorganisms-08-01895-s001.pdf]

Supplementary material

Table S1: Carbohydrate metabolism of lactic acid bacteria isolated from sourdough evaluated by OD_{620} measurements.

Isolat-code	Species	D-(-)-fructose	D-(+)-glucose	D-(+)-maltose	maltodextrine	D-(+)-raffinose	sucrose
TS1.8	*Enterococcus durans*	++	+++	+++	+	+	++
TS2.7	*Enterococcus durans*	++	++	++	++	+	+
TS3.7	*Enterococcus faecium*	++	++	++	++	+	++
TS1.7	*Enterococcus hirae*	+++	+++	+++	++	++	++
TS2.4	*Enterococcus hirae*	++	++	++	++	++	++
TS3.11	*Enterococcus hirae*	++	++	++	++	+	++
TS4.8	*Enterococcus hirae*	++	++	++	++	+	-
TS3.16	*Enterococcus mundtii*	++	++	++	++	+	++
TS3.5	*Enterococcus mundtii*	++	++	++	++	+	++
TS4.1	*Enterococcus mundtii*	++	++	++	++	-	++
S13.18	*Levilactobacillus brevis*	+++	+++	+++	+	-	-
S14.3	*Levilactobacillus brevis*	+++	+++	+++	+	-	-
S3.5	*Levilactobacillus brevis*	+++	+++	+++	-	-	+
S4.13	*Levilactobacillus brevis*	++	+++	+++	-	-	-
S4.5	*Levilactobacillus brevis*	+++	+++	++	+	-	+
S4.7	*Levilactobacillus brevis*	++	+++	+++	-	-	-
S5.14	*Levilactobacillus brevis*	++	+++	+++	+	-	+
S5.15	*Levilactobacillus brevis*	++	+++	+++	+	-	+
S5.2	*Levilactobacillus brevis*	++	+++	+++	+	-	+
S5.4	*Levilactobacillus brevis*	++	+++	+++	+	+	+
S5.9	*Levilactobacillus brevis*	++	+++	+++	+	-	+
S6.13	*Levilactobacillus brevis*	++	+++	+++	+	+	+
S6.8	*Levilactobacillus brevis*	++	+++	+++	-	-	-
S4.16	*Loigolactobacillus coryniformis*	+++	+++	+++	+++	++	+
S4.20	*Loigolactobacillus coryniformis*	+++	+++	++	+	+++	+++
S4.21	*Loigolactobacillus coryniformis*	+++	+++	+	+	+++	++
S4.23	*Loigolactobacillus coryniformis*	+++	+++	+++	+	-	+++
S4.3	*Loigolactobacillus coryniformis*	++	+++	++	+	+++	+++
S4.4.1	*Loigolactobacillus coryniformis*	+++	+++	++	+	+++	+++
S4.4.2	*Loigolactobacillus coryniformis*	+++	+++	+++	+	-	-
S4.9	*Loigolactobacillus coryniformis*	+++	+++	++	+	+++	+++
S6.17	*Loigolactobacillus coryniformis*	+++	+++	++	+	+++	+++
S6.19	*Loigolactobacillus coryniformis*	++	+++	++	-	+++	++
S6.9.1	*Loigolactobacillus coryniformis*	+++	+++	+++	+	+	+
S4.14	*Latilactobacillus curvatus*	++	++	++	+	-	+++
S4.15	*Latilactobacillus curvatus*	++	++	++	+	-	++
S4.25	*Latilactobacillus curvatus*	++	++	++	+	-	++
S5.22	*Latilactobacillus curvatus*	++	++	++	+	-	++
S5.7.1	*Latilactobacillus curvatus*	+++	+++	+++	+++	+++	+++
S6.15	*Latilactobacillus curvatus*	+++	+++	++	+	-	+
TS3.10	*Latilactobacillus curvatus*	++	++	++	+	-	++
TS3.18	*Latilactobacillus curvatus*	+++	++	++	+	-	+++
TS3.19	*Latilactobacillus curvatus*	+++	++	++	+	-	+++
TS3.20	*Latilactobacillus curvatus*	+++	++	++	+	-	++
TS3.4	*Latilactobacillus curvatus*	+++	++	++	+	-	++
TS3.8	*Latilactobacillus curvatus*	++	+++	++	+	-	++
TS3.9	*Latilactobacillus curvatus*	+	+++	++	+	++	++
TS4.11	*Latilactobacillus curvatus*	+++	+++	+++	++	+	++
TS4.12	*Latilactobacillus curvatus*	+++	+++	++	+	-	++
TS4.14	*Latilactobacillus curvatus*	+++	++	++	-	-	++
TS4.17	*Latilactobacillus curvatus*	+++	++	++	+	-	++
TS4.18	*Latilactobacillus curvatus*	+++	++	++	+	-	++
TS4.2	*Latilactobacillus curvatus*	+++	++	++	+	-	+++
TS4.3	*Latilactobacillus curvatus*	+++	+++	++	+	-	+++
TS4.6	*Latilactobacillus curvatus*	+++	++	++	+	-	++

TS4.7	*Latilactobacillus curvatus*	+++	++	++	-	-	+++
S14.1	*Levilactobacillus hammesii*	+++	++	+++	+	-	-
S7.10	*Companilactobacillus kimchii*	-	+++	+	+	-	-
S3.10	*Lentilactobacillus kisonensis*	+++	+++	+++	+	++	-
S3.4	*Lentilactobacillus kisonensis*	++	+++	+++	+	++	+
S3.9	*Lentilactobacillus kisonensis*	++	+++	+++	+	++	++
S3.1	*Lentilactobacillus otakiensis*	++	+++	-	-	-	+++
S3.11	*Lentilactobacillus otakiensis*	++	-	++	++	-	-
S3.15	*Lentilactobacillus otakiensis*	++	++	+++	++	-	++
S2.16	*Lentilactobacillus parabuchneri*	+++	+++	+++	-	+	+++
S2.9	*Lentilactobacillus parabuchneri*	++	+++	+++	-	+	+++
S2.21	*Lacticaseibacillus paracasei*	+++	+++	+	-	+	++
S8.13	*Lacticaseibacillus paracasei*	+++	+++	+++	++	+	+++
S8.21	*Lacticaseibacillus paracasei*	+++	+++	+++	-	-	+++
S8.24	*Lacticaseibacillus paracasei*	+++	+++	++	-	-	++
S8.3	*Lacticaseibacillus paracasei*	+++	+++	+++	-	-	++
S8.8	*Lacticaseibacillus paracasei*	+++	+++	++	+	-	++
S9.11	*Lacticaseibacillus paracasei*	+++	++	++	+	+	+++
S9.15	*Lacticaseibacillus paracasei*	+++	+++	+++	++	++	++
S9.18	*Lacticaseibacillus paracasei*	+++	+++	+++	++	-	++
S9.20	*Lacticaseibacillus paracasei*	+++	+++	+++	++	++	+++
S9.24	*Lacticaseibacillus paracasei*	+++	+++	++	+	-	++
S9.3	*Lacticaseibacillus paracasei*	+++	+++	+++	-	-	++
S9.8	*Lacticaseibacillus paracasei*	++	++	+++	-	-	++
S7.12	*Companilactobacillus paralimentarius*	-	+++	-	-	-	-
S7.14	*Companilactobacillus paralimentarius*	-	+++	-	-	-	-
S7.3	*Companilactobacillus paralimentarius*	-	+++	-	-	-	-
S7.5	*Companilactobacillus paralimentarius*	-	+++	-	+	-	-
S7.6	*Companilactobacillus paralimentarius*	-	+++	-	-	-	-
S7.8	*Companilactobacillus paralimentarius*	-	+++	-	-	-	-
S8.18	*Schleiferilactobacillus perolens*	+++	+++	+++	++	++	+++
S10.12	*Lactiplantibacillus plantarum*	++	+++	+++	++	+++	+++
S10.13	*Lactiplantibacillus plantarum*	+++	+++	+++	+++	+++	+++
S10.15	*Lactiplantibacillus plantarum*	++	+++	+++	+++	+++	+++
S10.19	*Lactiplantibacillus plantarum*	+++	+++	+++	+++	+++	+++
S10.2	*Lactiplantibacillus plantarum*	+++	+++	+++	++	+++	+
S10.9	*Lactiplantibacillus plantarum*	+++	+++	+++	+++	+++	+++
S13.13	*Lactiplantibacillus plantarum*	++	+++	+++	++	-	++
S13.8.2	*Lactiplantibacillus plantarum*	++	+++	+++	++	++	++
S18.5	*Lactiplantibacillus plantarum*	+++	+++	+++	++	+++	+++
S4.10	*Lactiplantibacillus plantarum*	+++	+++	+++	+++	+++	+++
S4.11	*Lactiplantibacillus plantarum*	+++	+++	+++	+++	+++	+++
S4.17	*Lactiplantibacillus plantarum*	+++	+++	+++	+++	+++	+++
S4.18	*Lactiplantibacillus plantarum*	+++	+++	+++	+++	+++	+++
S4.2	*Lactiplantibacillus plantarum*	+++	+++	+++	+++	+++	+++
S4.28	*Lactiplantibacillus plantarum*	+++	+++	+++	+++	+++	+++
S4.29	*Lactiplantibacillus plantarum*	+++	+++	+++	+++	+++	+++
S4.6	*Lactiplantibacillus plantarum*	+++	+++	+++	+++	+++	+++
S4.8	*Lactiplantibacillus plantarum*	+++	+++	+++	+++	+++	+++
S5.1	*Lactiplantibacillus plantarum*	+++	+++	+++	+++	+++	+++
S5.11	*Lactiplantibacillus plantarum*	+++	+++	+++	+++	+++	+++
S5.12	*Lactiplantibacillus plantarum*	+++	+++	+++	+++	+++	+++
S5.13	*Lactiplantibacillus plantarum*	+++	+++	+++	+++	+++	+++
S5.16	*Lactiplantibacillus plantarum*	+++	+++	+++	++	+++	+++
S5.5	*Lactiplantibacillus plantarum*	+++	+++	+++	+++	+++	+++
S6.11	*Lactiplantibacillus plantarum*	+++	+++	+++	+++	+++	+++
S6.14	*Lactiplantibacillus plantarum*	+++	+++	+++	+++	+++	+++
S6.18	*Lactiplantibacillus plantarum*	+++	+++	+++	+++	+++	+++

S6.2	*Lactiplantibacillus plantarum*	+++	+++	+++	+++	+++	+++
S6.3	*Lactiplantibacillus plantarum*	+++	+++	+++	+++	+++	+++
S6.5	*Lactiplantibacillus plantarum*	+++	+++	+++	+++	+++	+++
S6.6	*Lactiplantibacillus plantarum*	+++	+++	+++	+++	+++	+++
S7.4	*Lactiplantibacillus plantarum*	-	+++	+++	-	-	-
S2.25	*Limosilactobacillus pontis*	+	++	+++	-	+	-
S4.12	*Latilactobacillus sakei*	+++	+++	-	-	-	+++
S4.19	*Latilactobacillus sakei*	+++	+++	-	+	-	+++
S4.22	*Latilactobacillus sakei*	+++	+++	-	-	-	+++
S7.1	*Fructilactobacillus sanfranciscensis*	-	+++	+++	-	-	-
S7.2	*Fructilactobacillus sanfranciscensis*	-	+++	+++	-	+	-
S7.7	*Fructilactobacillus sanfranciscensis*	-	+++	+++	-	+	-
S7.9	*Fructilactobacillus sanfranciscensis*	-	+++	+++	-	+	+
TS6.7	*Fructilactobacillus sanfranciscensis*	-	+++	+++	+	-	-
TS7.3	*Fructilactobacillus sanfranciscensis*	-	-	+	-	-	-
S4.24	*Levilactobacillus senmaizukei*	++	+++	+++	+	-	+
S4.27	*Levilactobacillus senmaizukei*	++	+++	+++	-	-	-
S5.10	*Levilactobacillus senmaizukei*	++	+++	+++	+	-	-
S5.17	*Levilactobacillus senmaizukei*	++	+++	+++	+	-	+
S5.18	*Levilactobacillus senmaizukei*	++	+++	+++	+	-	+
S5.21	*Levilactobacillus senmaizukei*	++	+++	+++	+	-	+
S5.23	*Levilactobacillus senmaizukei*	++	+++	+++	-	-	-
S5.8	*Levilactobacillus senmaizukei*	++	+++	+++	+	-	+
S6.16	*Levilactobacillus senmaizukei*	++	+++	+++	+	+	+
S6.22	*Levilactobacillus senmaizukei*	+++	+++	+++	+	+	+
S6.4	*Levilactobacillus senmaizukei*	+++	+++	+++	-	-	-
S13.10	*Levilactobacillus spicheri*	+++	++	+++	-	-	-
S4.26	*Levilactobacillus spicheri*	+++	+++	+++	++	-	-
S6.1.1	*Paucilactobacillus vaccinostercus*	-	++	-	-	-	-
S6.1.2	*Paucilactobacillus vaccinostercus*	-	+++	+++	-	-	-
S6.20	*Paucilactobacillus vaccinostercus*	-	+++	+++	-	-	-
S6.7	*Paucilactobacillus vaccinostercus*	-	+++	+++	+	+	-
S7.11	*Lactiplantibacillus xiangfangensis*	+++	+++	++	++	++	+++
S7.13	*Lactiplantibacillus xiangfangensis*	+++	+++	++	++	+	++
TS2.6	*Leuconostoc citreum*	++	++	++	+	+	++
TS2.8	*Leuconostoc citreum*	++	++	++	++	-	++
TS4.20	*Leuconostoc citreum*	++	++	+++	+	+++	+++
TS1.6	*Leuconostoc lactis*	+	++	++	+	++	++
S5.6	*Pediococcus parvulus*	-	-	-	-	-	-
S1.19	*Pediococcus pentosaceus*	+++	+++	+++	+	-	+
S1.20	*Pediococcus pentosaceus*	+++	+++	+++	+	-	+
S10.10	*Pediococcus pentosaceus*	-	+++	+++	+++	-	-
S10.11	*Pediococcus pentosaceus*	++	++	+++	++	++	++
S5.19	*Pediococcus pentosaceus*	+++	+++	+++	-	-	-
S5.3	*Pediococcus pentosaceus*	++	+++	+++	+	-	+
TS1.4	*Pediococcus pentosaceus*	+++	+++	+++	-	+	+
TS3.1	*Pediococcus pentosaceus*	+++	+++	+++	-	+++	+++
TS3.12	*Pediococcus pentosaceus*	+++	+++	+++	++	+	+
TS3.14	*Pediococcus pentosaceus*	+++	+++	+++	++	+	+++
TS3.15	*Pediococcus pentosaceus*	+++	+++	+++	++	+	+++
TS3.2	*Pediococcus pentosaceus*	+++	+++	+++	+	+++	+++
TS3.3	*Pediococcus pentosaceus*	+++	+++	+++	+	+++	+++

TS3.6	*Pediococcus pentosaceus*	+++	+++	+++	-	+++	+++
TS4.10	*Pediococcus pentosaceus*	+++	+++	+++	++	+	+
TS4.15	*Pediococcus pentosaceus*	+++	+++	+++	++	+++	+++
TS4.16	*Pediococcus pentosaceus*	+++	+++	+++	+	+++	+++
TS4.19	*Pediococcus pentosaceus*	+++	+++	+++	+	+++	+++
TS4.4	*Pediococcus pentosaceus*	+++	+++	+++	+	+	+
TS4.5	*Pediococcus pentosaceus*	+++	+++	+++	+	+	+
TS4.9	*Pediococcus pentosaceus*	+++	+++	+++	+	+	+
S6.9.2	*Streptococcus salivarius*	-	+++	++	-	-	-
S1.1	*Weissella cibaria*	++	++	++	+	-	++
S1.5	*Weissella cibaria*	+	+++	++	-	-	++
S1.7	*Weissella cibaria*	+	++	+++	+	-	++
S10.4	*Weissella cibaria*	++	++	+++	+	-	++
TS1.1	*Weissella cibaria*	++	+++	+++	+	-	+++
TS1.3	*Weissella cibaria*	+	+++	+++	+	-	++
TS1.5	*Weissella cibaria*	++	+++	+++	-	-	++
S1.2	*Weissella viridescens*	-	-	-	-	-	-
S1.16	*Weissella viridescens*	n.a.	n.a.	n.a.	n.a.	n.a.	n.a.
S2.3	*Weissella viridescens*	-	-	-	-	-	-

+++ very strong growth; ++ strong growth; + weak growth; - no growth; n.a. not analyzed

Table S2. LAB isolates used for genomic strain differentiation by repetitive element PCR.

Species	Number of isolates	Isolat-codes
Enterococcus durans	2	TS1.8, TS2.7
Enterococcus faecium	1	TS3.7
Enterococcus hirae	4	TS1.7, TS2.4, TS3.11, TS4.8
Enterococcus mundtii	3	TS3.16, TS3.5, TS4.1
Levilactobacillus brevis	13	S13.18, S14.3, S3.5, S4.13, S4.5, S4.7, S5.14, S5.15, S5.2, S5.4, S5.9, S6.13, S6.8
Loigolactobacillus coryniformis	10	S4.16, S4.20, S4.21, S4.23, S4.4.1, S4.4.2, S4.9, S6.17, S6.19, S6.9.1
Latilactobacillus curvatus	21	S4.14, S4.15, S4.25, S5.22, S5.7.1, S6.15, TS3.10, TS3.18, TS3.19, TS3.20, TS3.4, TS3.8, TS3.9, TS4.11, TS4.12, TS4.17, TS4.18, TS4.2, TS4.3, TS4.6, TS4.7
Levilactobacillus hammesii	1	S14.1
Companilactobacillus kimchii	1	S7.10
Lentilactobacillus kisonensis	3	S3.10, S3.4, S3.9
Lentilactobacillus otakiensis	3	S3.1, S3.11, S3.15
Lentilactobacillus parabuchneri	1	S2.16, S2.9
Lacticaseibacillus paracasei	13	S2.21, S8.13, S8.21, S8.24, S8.3, S8.8, S9.11, S9.15, S9.18, S9.20, S9.24, S9.3, S9.8
Companilactobacillus paralimentarius	6	S7.12, S7.14, S7.3, S7.5, S7.6, S7.8
Schleiferilactobacillus perolens	1	S8.18
Lactiplantibacillus plantarum	32	S10.12, S10.13, S10.15, S10.19, S10.2, S10.9, S13.13, S13.8.2, S18.5, S4.10, S4.11, S4.17, S4.18, S4.2, S4.28, S4.29, S4.6, S4.8, S5.1, S5.11, S5.12, S5.13, S5.16, S5.5, S6.11, S6.14, S6.18, S6.2, S6.3, S6.5, S6.6, S7.4
Limosilactobacillus pontis	1	S2.25
Latilactobacillus sakei	3	S4.12, S4.19, S4.22
Fructilactobacillus sanfranciscensis	6	S7.1, S7.2, S7.7, S7.9, TS6.7, TS7.3
Levilactobacillus senmaizukei	11	S4.24, S4.27, S5.10, S5.17, S5.18, S5.21, S5.23, S5.8, S6.16, S6.22, S6.4
Levilactobacillus spicheri	3	S13.10, S4.26
Paucilactobacillus vaccinostercus	4	S6.1.1, S6.1.2, S6.20, S6.7
Lactiplantibacillus xiangfangensis	2	S7.11, S7.13
Leuconostoc citreum	3	TS2.6, TS2.8, TS4.20
Leuconostoc lactis	1	TS1.6
Pediococcus pentosaceus	21	S1.19, S1.20, S10.10, S10.11, S5.19, S5.3, TS1.4, TS3.1, TS3.12, TS3.14, TS3.15, TS3.2, TS3.3, TS3.6, TS4.10, TS4.15, TS4.16, TS4.19, TS4.4, TS4.5, TS4.9
Streptococcus salivarius	1	S6.9.2
Weissella cibaria	7	S1.1, S1.5, S1.7, S10.4, TS1.1, TS1.3, TS1.5

Table S3: Correlation coefficients of growth applying different carbohydrate sources on the inhibition of fungal activity.

	**Correlation coefficients**				
**sole carbohydrate source**	***Aspergillus flavus* MUCL11945**	***Fusarium graminearum* MUCL43764**	***Aspergillus fumigatus***	***Aspergillus brasiliensis* DSM1988**	***Penicillium roqueforti* DSM1079**
**Glucose**	-0.087	-0.509	0.727*	0.958**	0.995**
**Fructose**	-0.086	-0.509	0.731*	0.960**	0.995**
**Maltose**	-0.056	-0.532	0.693	0.934**	0.990**
**Sucrose**	-0.086	-0.508	0.734*	0.961**	0.995**
**Maltodextrin**	-0.153	-0.488	0.677	0.945**	0.992**
**Raffinose**	-0.125	-0.489	0.727*	0.965**	0.994**

** correlation is highly significant at the p level of 0.01

* correlation is significant at the p level of 0.05

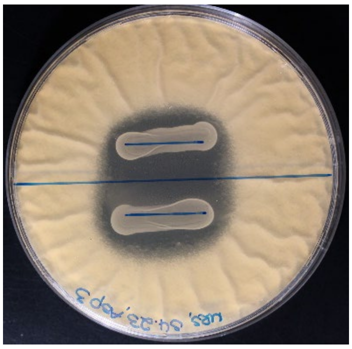

(**a**)

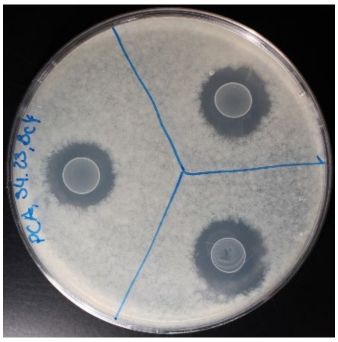

(**b**)

Figure S1. Evaluation of antimicrobial potential of *Loigolactobacillus coryniformis* S4.23 against (a) *Aspergillus fumigatus* using the cultural overlay assay, and (b) *Bacillus cereus* DSM31 by using the spot-on-the-lawn technique.
